# The partner’s experiences of childbirth in countries with a highly developed clinical setting: a scoping review

**DOI:** 10.1186/s12884-022-05014-1

**Published:** 2022-10-03

**Authors:** Nadine Schmitt, Sabine Striebich, Gabriele Meyer, Almuth Berg, Gertrud M. Ayerle

**Affiliations:** 1 Medical Faculty, Institute for Health and Nursing Science, Interdisciplinary Center for Health Sciences, Martin Luther University Halle-Wittenberg, Magdeburger Straße 8, 06112 Halle (Saale), Germany; 2 Department of Nursing and Management, Hamburg University of Applied Sciences, Berliner Tor 5, 20099 Hamburg, Germany

**Keywords:** Partner, Support person, Midwifery, Childbirth experience, Fathers, Review

## Abstract

**Background:**

In Western countries, it is common practice for a woman to be supported by a trusted person during childbirth, usually the other parent. Numerous studies have shown that this has a positive effect both on the woman’s satisfaction with the birth process and on physical outcomes. However, there is little research on the birth experience of partners and their wellbeing. The aim of this review is to summarise the existing literature on partner experience, consider its quality and identify the underlying themes.

**Methods:**

Both a systematic literature search in three databases and a manual search were conducted, for qualitative, quantitative, and mixed-methods studies from Western countries examining the experiences of partners present at a birth.

**Results:**

A total of 35 studies were included. Only one study included same-sex partners (the other studies addressed fathers’ experiences only) and only one validated questionnaire examining partners’ birth experiences was identified. Four major themes were found to influence partners’ birth experiences: (1) intense feelings, (2) role of support, (3) staff support, and (4) becoming a father.

**Conclusions:**

Partners may feel very vulnerable and stressed in this unfamiliar situation. They need emotional and informal support from staff, want to be actively involved, and play an important role for the birthing woman. To promote good attachment for parents, systematic exploration of the needs of partners is essential for a positive birth experience. Because of the diversity of family constellations, all partners should be included in further studies, especially same-sex partners.

**Supplementary Information:**

The online version contains supplementary material available at 10.1186/s12884-022-05014-1.

## Background

Until the 1980’s, childbirth was considered “women’s business”. For this reason, as well as because of strict hygiene regulations, partners were not desired or encouraged to attend the birth [1]. It has only been in the last 40 years that support from a trusted person has been encouraged as part of family-oriented childbirth [2]. The partner’s presence at birth has increased significantly, at least in Western countries [3].

The majority of partners want to participate in the birth of their child and support the women [4]. Partners describe the birth of their child as a moment of realisation, a transformation into becoming a father [5]. Partners who participated in the birth feel they have “grown” into fatherhood [6] and report higher attachment scores than partners who did not participate in the birth [7]. The more involved the partner is during childbirth, the stronger the bond with the baby will be [8]. Gettler et al. suggest that birth represents a phase in which paternal psychobiology responds to fathers’ new experiences in interacting with their newborns [9]. They found that first-time fathers’ oxytocin levels were higher after holding their newborn for the first time than before. They also found that fathers played more with their infants after birth when their oxytocin levels increased and testosterone decreased. Thus, stronger attachment is associated with greater involvement. Fathers’ involvement during childhood has positive effects on children’s development, such as physical health and social skills [10]. Therefore, strong attachment is likely to have a lasting positive impact on the family and society as a whole [11].

In contrast to the body of knowledge outlined above, partners often receive little attention and are not always involved during childbirth [12, 13]. They report feeling excluded and unsupported by the healthcare system during pregnancy and childbirth [14]. If the partner does not experience support and does not feel included, he or she will not be able to adequately support the woman [15].

Lack of communication between partners and medical staff and the feeling of exclusion can lead to a negative birth experience for the partner [16]. A negative birth experience can affect fathers’ mental health [17]. It is associated with an increased risk of postpartum depression, for example [18], and can even lead to symptoms of post-traumatic stress disorder (PTSD) [19]. A negative birth experience and PTSD can in turn lead to disrupted attachment between parent and child and be associated with negative parenting outcomes [18].

While in low-income countries very low caesarean section rates with a high maternal mortality risk are reported, in high-income countries caesarean section rates have risen sharply since the 1970s [20]. In many highly developed Western countries, an intervention-rich obstetric care is currently practised in hospitals, where excessive and non-evidence-based use of interventions can be observed [21]. A high degree of medicalisation and high rates of intervention, with one in three children born by caesarean section [22], characterise clinical obstetric care in Germany. It has been indicated that less than 10% of low-risk women birthing in hospital in Germany experience a natural birth without any invasive interventions [23]. Despite a lack of or inconclusive evidence regarding interventions, the physiological birth process is frequently intervened in [24]. This is countered by the fact that childbirth is one of the most important personal experiences in many women’s lives and it is important for many women to experience a physiologic birth process [25].

In 2018, the World Health Organization published a guideline with evidence-based recommendations for low-risk births [26]. The goal is to avoid unnecessary interventions and therefore promote physiological births. Social support during childbirth is an important component of a natural birth. A positive birth experience is related to the woman being accompanied by someone she knows and trusts [27]. The WHO clarifies that every woman has the right to have a companion of her choice present during childbirth [28]. A Cochrane review concludes that the presence of a companion has a positive effect on the birth process: Women who received continuous support were more likely to have a spontaneous vaginal birth and were less likely to require intrapartum analgesia, their labour was shorter, and they were more satisfied with the birth process [29].

The companionship of a close person has been shown to be important for birthing women [30–32]. Companions provide emotional, psychological and physical support during labour, which contributes to a more positive birth experience [27, 32–34]. Specifically, the presence of a partner promotes trust and safety, can alleviate pain and feelings of loneliness, create emotional and physical wellbeing [35], contribute to self-confidence and strength in coping with childbirth [32], and promote women’s sense of control in labour [36].

Nowadays, in most Western countries, it is common for expectant fathers to be present in the birthing room and to actively participate in their partner’s labour and birth [32]. While the subjective feelings of the women giving birth are increasingly addressed, research on partners’ experiences of childbirth is still quite scarce [11, 37]. Men are seen as companions during pregnancy and birth, but are not treated as individuals with their own needs [38]. If we want to promote secure attachment between both parents and their child, we need to create conditions that support the development of partners’ attachment hormones too. This includes a positive birth experience for the partner and an understanding of what partners need to achieve that. According to Nielsen & Overgaard, to support true family development involvement, the individual needs of the partner should be explored [39]. We also need to identify factors that lead to a negative birth experience so as to be able to preventively protect partners’ mental health.

This scoping review therefore aims to identify, review and synthesise the literature on the experiences of partners during childbirth in clinical settings in Western countries in order to identify the different themes and subthemes, which influence these experiences.

## Methods

We used the design of a scoping review since it maps the key concepts underlying an area of research and the main sources and types of available evidence [40]. Scoping reviews typically address broad questions and may include a range of methods. This is in contrast to a systematic review, where the research question is narrowly defined and the included studies are critically appraised. For this study, a scoping review was deemed most appropriate for mapping the literature and identify the main themes related to partners’ birth experiences. This review follows the five steps of Arksey & O’Malley’s framework [40]: 1. Identifying the research question, 2. Identifying relevant studies, 3. Study selection, 4. Charting the data, 5. Collating, summarising and reporting the results. In addition to these five steps, the Preferred Reporting Items for Systematic reviews and Meta-Analyses extension for Scoping Reviews (PRISMA_ScR) Checklist was used to ensure completeness of the procedure (Additional File 1) [41]. To reflect the heterogeneity of the included studies and their methodological quality, we have included a critical appraisal of these studies in this scoping review.

### Identifying the research question

What are the experiences of partners attending the birth of their child in clinical settings in Western countries?

Although heteronormative relationships are the most common type of relationship and thus the father is the most common companion, diverse constellations of relationships exist. For this reason, we searched for the experience of partners in general. However, with the exception of one study, we found only studies that examined the experiences of fathers. For this reason, we refer to fathers in this review.

### Identifying relevant studies

The beginnings of partner birth attendance 1980s are not comparable to today`s birth attendance. For this reason, and due to societal changes regarding the inclusion of fathers and equal parenting, especially from the turn of the millennium onwards, we conducted a review of the literature published after 2000. Initial searches were conducted in Medline via PubMed and CINAHL and in the midwifery digest MIDIRS in May 2021. To find additional relevant studies, we manually searched the Internet and used citation tracking. Since this work is a preliminary work for the development of a German language questionnaire for partners during childbirth, we were strongly interested in what publications have appeared in Germany on this topic. For this reason we manually reviewed professional journals in the field of maternity care published in Germany via database Thieme publisher. The search terms (Table 1) were piloted (i.e., terms like ‘labour’ that yielded too many hits were searched in the title only) and adjusted according to the databases. To achieve efficient search results, we used truncations according to the requirements of the databases and MeSH terms for Medline. For example, the search string for PubMed was as follows:

**Table 1:** Search terms used^a^

Population	Context	Outcome
labour companionship	labor	well-being
companion*	labour	wellbeing
father*	birth*	welfare
husband*	childbirth	emotion*
birth partner*	parturition	affectiv*
support person		psychologic*
spouse*		experienc*

^a^ based on the PICO scheme; the words marked with * are truncations

(“labour companionship”[tiab] OR companion*[tiab] OR father*[tiab] OR husband*[tiab] OR “birth partner*”[tiab] OR “support person” OR spouse* OR spouses[MeSH]) AND (well-being[tiab] OR wellbeing[tiab] OR welfare[tiab] OR emotion*[tiab] OR affectiv*[tiab] OR psychologic*[tiab] OR experienc*[tiab]) AND (labour[ti] labor[ti] OR birth*[ti] OR childbirth[ti] OR parturition[ti] OR parturition[MeSH]).

### Study selection

We considered as eligible all qualitative, quantitative and mixed-method studies which examined the birth experiences of partners in the clinical setting, whether or not it was their first attendance at a birth. Any published language was included. Table 2 lists the inclusion and exclusion criteria.

**Table 2:** Inclusion and exclusion criteria

	Inclusion criteria	Exclusion criteria
Participants	People attending the birth for the first or repeated time	Focus on experiences of the birthing woman Focus on specific group of people or age range Doulas
Exposure	Focus on overall birth experience	Focus on prenatal or postnatal experience or mental disorder Focus on antenatal classes Focus on transition to fatherhood
Setting	Clinical setting	Homebirth or birth centre
Birth mode	All term birth modes as long as vaginal birth is included	Focus on caesarean or instrumental birth Focus on premature birth Focus on birth with complications or traumatic birth
Countries	North-, South- and West-Europe, Canada, Australia, New Zealand	Eastern Europe, North and South America, developing and emerging countries
Time period	Published after 2000	Published before 2000
Language	All languages	-
Type of studies	Quantitative, qualitative and mixed-method	Editorials, reports, grey literature; reviews

For comparability, studies were included from Western countries, covering Northern, Western and Southern European countries, Australia, New Zealand, and Canada (for a detailed list of related countries see: https://unstats.un.org/unsd/methodology/m49/). Since obstetric care in Eastern European countries and North and South America differs greatly from that in Germany, studies from these countries were not included, nor were studies from developing countries.

### Charting the data

Two independent researchers (NSch and SStr) extracted the data. First, all titles and abstracts were checked for inclusion, and the included studies were then reviewed for relevance using the full text. Disagreements between researchers were resolved through discussion and consensus. If necessary, the opinion of a third researcher (GA) was obtained. Researchers extracted information on study characteristics using a piloted data extraction form. Data extraction included information about the authors and year, the country in which the study was conducted, a description of the participants, the birth modes included in the study, information about which method was used to examine the birth experience and a summary of the results found.

### Collating, summarising and reporting the results

Due to heterogeneity in study design and quality, various birth experience themes were categorised and described. Some sources addressed experiences during pregnancy or postpartum in addition to the birth experience. Here, only evidence related to the birth experience was extracted. If both mothers and fathers were interviewed in the publication, we considered only the experiences of fathers, also in comparison to mothers, whenever possible.

In order to identify recurring themes regarding birth experiences, the main results of all included studies were first recorded in a comprehensive table. Then, similarities and differences in the data were systematically identified by rereading the studies’ main results. Obvious recurring themes were first colour coded. Subsequently, each main result was assigned to a theme. Four themes common across all studies were identified.

Because we combined qualitative, quantitative and mixed-methods studies, we used the Mixed Methods Appraisal Tool (MMAT) to assess the quality of the studies [42]. Screening questions were answered to determine the empirical nature of the study and to assess the key features of its methodology. The first author (NSch) assessed all studies, while co-authors (GA, AB, GM, SStr), acting as independent second reviewers, assessed a subset of studies each. Disagreements were resolved through discussion between the authors.

## Results

### Search outcomes and study characteristics

**Fig. 1:**
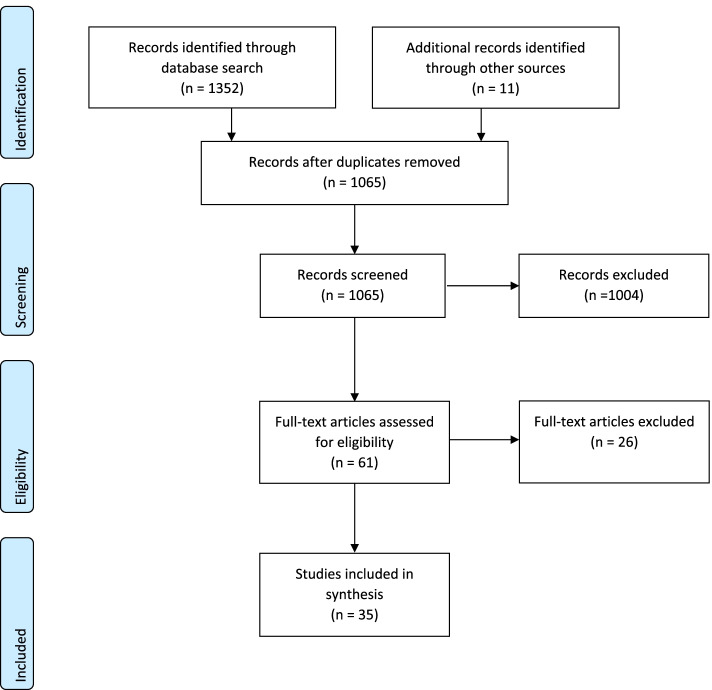
Literature selection flowchart

The flow chart of the literature selection (Fig. 1) provides an overview of the search results. After removing duplicates, 1054 hits (1003 + 51) were identified in the Medline, CINAHL and MIDIRS databases and then screened. Citation tracking and other search sources (e.g., asking colleagues) yielded 11 additional hits. Of these, 61 abstracts related to fathers’ experiences. The scoping review includes 35 studies that met the inclusion criteria. 13 used a qualitative method approach, 18 used a quantitative approach and four a mixed-method approach. In some studies, participants were first-time fathers. In other studies, the experiences of fathers who did not have their first child but had another child were examined. Seven studies also examined the experiences of the women giving birth [43–49]. Another examined the experiences of the mother and sisters of the women giving birth [50]. As mentioned above, only one study included same-sex partners in addition to fathers [44]. The time span of postpartum follow-up ranged from 24 h after birth to one year after birth. Some studies did not specify at what time after birth the data collection took place.

The majority of the qualitative studies used open or semi-structured interviews, two of them in combination with observation. In the included mixed-method studies, data were collected mainly through closed- and open-ended questions. Most quantitative studies were conducted with self-developed questionnaires. In one case the Salmon’s Item List was adapted to men [51], and in another, the rate of PTSD was measured using established instruments [52]. One study combined the results of their questionnaire with data collected using the State-Trait-Anxiety Inventory (STAI) during childbirth [11]. In the period from 2000 to 2021, only one validated questionnaire (the First-time Father Questionnaire) was found to measure birth experiences of partners, and two of the included studies referred to it [53, 54]. Additional File 2 shows the assessment of methodological quality of the included studies. Here it can be seen that the majority of the qualitative studies have good validity. The quantitative studies, on the other hand, are only of moderate to low validity. This is mainly due to the fact that the studies used poorly validated questionnaires to investigate the birth experience of partners. Another issue that affects validity is that many quantitative studies contain incomplete outcome data.

### Findings – thematic areas of birth experience

Four main themes relate to fathers’ experiences of clinical birth: Intense feelings, the role of support, staff support, and the becoming a father. Table 3 provides an overview of the included studies.

**Table 3:** Characteristics of the included studies

Authors, Year	Country	Sample	Birth modes^a^	Study design
Awad & Bühling, 2011 [55]	Germany	86 first-time and multiple fathers	All	Quant
Bäckström & Hertfelt Wahn, 2011 [56]	Sweden	10 first-time fathers	Vaginal	Qual
Bélanger-Lévesque et al., 2014 [44]	Canada	200 first-time and multiple parents	All	Quant
Bradley et al., 2008 [52]	UK	199 first-time and multiple fathers	not specified	Quant
Capogna et al., 2007 [11]	Italy	243 fathers	Vaginal	Quant
Chan & Paterson-Brown, 2002 [45]	UK	114 fathers and 112 mothers	All	Quant
Eggermont et al., 2017 [57]	Belgium	72 first-time or multiple fathers	Vaginal	Quant
Erlandsson & Lindgren, 2009 [5]	Sweden	16 first-time and multiple fathers	All	Qual
Franzen et al., 2021 [54]	Switzerland & France	151 first-time fathers	All	Quant
Gawlik et al., 2015 [51]	Germany	88 first-time and multiple fathers	All	Quant
Harte et al., 2016 [50]	Australia	first-time father; mother and sisters of the birthing woman	not specified	Qual
Hildingsson et al., 2011 [58]	Sweden	595 first-time and multiple fathers	Normal vaginal	Quant
Howarth et al., 2019 [59]	New Zealand	155 first-time fathers	not specified	Mix
Johansson et al., 2012 [60]	Sweden	827 first-time and multiple fathers	All	Mix
Johansson & Hildingsson, 2013 [61]	Sweden	827 first time and multiple fathers	All	Quant
Johansson & Thies-Lagergren, 2015 [62]	Sweden	221 fathers	All	Mix
Johnson, 2002a [63]	UK	53 fathers	Normal vaginal	Quant
Johnson, 2002b [64]	UK	53 resp. 20 first-time and multiple fathers	Normal vaginal	Mix
Kopff-Landas et al., 2008 [49]	France	33 first-time parents	Vaginal	Qual
Köhne & Hellmers, 2015 [65]	Germany	12 fathers	Vaginal	Qual
Krulis et al., 2021 [66]	Austria	12 first-time fathers	no primary CS	Qual
Ledenfors & Berterö, 2016 [67]	Sweden	8 first-time fathers	Normal vaginal	Qual
Longworth & Kingdon, 2011 [68]	UK	11 first-time fathers	not specified	Qual
Longworth et al., 2021 [43]	UK	12 first and multiple parents	not specified	Qual
Moreau et al., 2009 [48]	France	33 first-time parents	Vaginal	Quant
Nystedt & Hildingsson, 2018 [46]	Sweden	928 first-time mothers and 818 first-time fathers	All	Quant
Porrett et al., 2013 [69]	Australia	163 first-time fathers	All	Quant
Premberg et al., 2011 [70]	Sweden	10 first-time fathers	Vaginal	Qual
Premberg et al., 2012 [53]	Sweden	200 first time fathers	All	Quant
Rosich-Medina & Shetty, 2007 [71]	United Kingdom	150 first-time fathers	All	Quant
Sapountzi-Krepia et al., 2015 [72]	Greece	228 first-time and multiple fathers	not specified	Qual
Sydow & Happ, 2012 [73]	Germany	30 first-time fathers	All	Qual
Tarlazzi et al., 2015 [74]	Italy	6 first-time fathers	Vaginal	Qual
Thies-Lagergren & Johansson, 2019 [47]	Sweden	209 couples	All	Quant
Vischer et al., 2020 [75]	Germany	318 first-time and multiple fathers	All	Quant

*Quant* Quantitative, *Qual* Qualitative, *Mix* Mixed-method, *CS* caesarean section

^a^normal vaginal birth was described by the authors as a physiological birth without instrumental assistance

#### Intense feelings

Almost all included studies report intense feelings among fathers. The most commonly reported feelings are anxiety, ranging from worry to fear, and helplessness [49, 53–55, 60, 67, 70, 71, 74, 75]. For fathers, dealing with women’s pain during birth is extremely challenging [49, 51, 55, 59, 66, 69, 72], especially when pain increases, something unexpected happens, and the couple is left alone [74]. Fathers worry about the health and life of both the woman and the baby [59, 60, 69, 73]. The inability to help her or to share the pain is one of the overwhelming memories [64] and leads to helplessness [65]. This helplessness can lead to feelings of panic [46, 56]. The greater the level of anxiety, the lower the satisfaction with the birth experience [11]. Higher levels of anxiety have been found to be associated with an unplanned pregnancy, feeling poorly prepared for labour and birth, a lower sense of control, and paternal history of mental health issues [52].

While some studies show that fathers who already have children are less fearful [60], Bradley et al. report higher levels of ‘intrusion’ and ‘avoidance’ (symptoms of PTSD) when fewer children and fewer births were experienced [52]. Regardless of the number of births experienced, Vischer et al. (2020) found that the PTSD symptom ‘intrusion’ was still prevalent in fathers six months after birth. The authors’ explanation is that the birth experience is still very present due to the memorable event. They did, however, find that not a single father met all the criteria for PTSD after experiencing the birth [75].

Fathers feel vulnerable and highly stressed in this unknown situation [63, 67] and report a variety of emotions. Gawlik et al. describe the birth experience for fathers as a multidimensional process, similar to mothers’ [51]. Fathers are unsure of how to act [70] and struggle with emotional distress [48, 74]. Feelings of lack of control [47, 60] are described, as are feelings of tension or guilt [73]. Stress levels are particularly high among fathers who felt pressured to be present at birth [63]. Fathers sometimes report ambivalent feelings: they want to be there, but at the same time are afraid of what they might see [74], or even have the impulse to flee [73]. In two studies, some fathers expressed fears about negative effects on their sex lives [55, 73]. While some studies report that fathers felt well prepared [45], in others all fathers stated that they were not really prepared for what was happening because they could not imagine it [67, 74]. Some fathers talk about the discrepancy between their expectations and the actual duration of the birth, in both directions [65, 66].

The environment also has an impact on fathers’ feelings. Men state that the unfamiliar environment in a hospital birthing room causes discomfort [64] or criticise the equipment, describing a lack of privacy and even seating [66]. Harte et al. conducted a single-case study to examine the influence of the hospital environment on the experiences of birth companions [50]. They found that support people felt disorientated, inhibited and hesitant in the environment, with the predominant feeling was ‘unbelonging’. They wanted to build a nest for the women, but felt foreign, uncomfortable and lacking in privacy. They felt that they had no control over the birthing room; the equipment frightened them and they found it disruptive.

Other factors affecting fathers’ feelings are the impacts of the birth process itself. Experiencing interventions or witnessing complications is perceived as stressful and difficult [65, 72]. Men whose partners adopt an upright position are more likely to have a positive birth experience and feel more comfortable and powerful than those where a horizontal birth position is adopted [62]. There were conflicting results regarding the use of analgesia in labour. In one study, men whose partners received analgesia perceived their presence as more necessary, helpful and relaxing. They felt more involved, less anxious and stressed [11]. In contrast, in the study by Bélanger-Lévesque et al., the use of epidural analgesia is a significant predictor of lower satisfaction [44]. Different modes of birth also affect fathers’ feelings differently, although the data are also inconsistent. Premberg et al. found that fathers were more worried when the child was born by caesarean section or instrumental birth than when a spontaneous vaginal birth was possible [53]. Rosich-Medina & Shetty and Johansson & Hildingsson report more negative feelings about emergency caesarean sections and instrumental births in fathers, than in those who witness vaginal birth or elective caesarean section [61, 71]. Bélanger-Lévesque et al. report lower satisfaction among fathers attending instrumental birth and primary caesarean section [44]. Chan & Paterson-Brown, however, report more negative feelings during a caesarean birth than during a normal or instrumental vaginal birth [45]. In contrast, Porrett et al. found no significant difference in fathers’ experiences between birth modes [69].

Despite these negative feelings, many of the included studies report positive overall experiences for fathers [44–49, 51, 58]. Most men report a desire to be present at a future birth and advise other men to attend [54, 55, 64, 75].

The studies comparing the experiences of fathers and mothers all concluded that the overall experience was the same for both parents, but that they differed on individual subthemes [44, 46–48]. While the fathers feel they were not supportive, mothers report the opposite. Women rate father involvement and support as more active and positive than men do [45, 47, 48]. Men hide their feelings from the woman giving birth so as not to worry her [70]. However, there are also findings that the fathers’ experiences are rated more negatively by mothers than by fathers themselves [45], which leads the authors to conclude that the men do not seem to have hidden their feelings from women.

The feelings reported by fathers also relate to the role they assumed during childbirth. Johnson report higher levels of stress in men who did not fulfil their expectations of the role [63].

#### Role of support

In five studies, the majority of men were found to have felt helpful and important in supporting the woman giving birth [49, 55, 67, 69, 75]. However, in four studies the opposite was found: men felt unable to meet the mother’s needs and did not believe that they had been supportive [47, 62, 63, 68]. Partners aim to provide comfort and protection [43, 50], for example, by withholding negative information or advocating for the woman during conflicts with staff [70]. They provide *emotional support* by being present and offering conversation, *physical support* by aiding different birthing positions or easing mobility, and *informational support* by mediating between staff and the woman [49, 65]. They try to be part of the process [67], want to be seen as one half of the birthing couple [56], but are sometimes described as being on the edge of events [68]. It can be difficult for the father to find his role, regardless of factors such as environment or staff [68]. Sometimes the role of the father is described as ‘just being there’ [68, 74]. Several studies report that men would like to be more involved [58, 62] and some even report feeling they have no role or are in the way [50, 63, 64]. When fathers feel involved in the birth process, they also feel more useful [69]. In Krulis et al. the majority of the fathers were satisfied with the role they played [66].

It is typical for the man to put his own needs aside and to hide own feelings [70]. The role of the support person is described as highly variable [66], depending on personality [74], and an individual partner’s assumed role can change during the birth process [50]. Tarlazzi et al. describe fathers as more engaged and active in the second stage of labour when pain is described as more active [74].

Studies attempting to classify different roles describe, for example, an *observer role* (distant and disinterested, passive or vigilant active observer role), a *carer role* (providing comfort and emotional support), an *intermediary role* (facilitating information sharing) and an *advocate role* (representing the woman’s needs by advocating for her) [68].

The environment influences the role of support people by either providing a place to be present and responsive to the woman’s needs or by preventing closeness [50]. Technology influences this, as do the presence of staff and the woman’s expectations and encouragement [43].

#### Staff support

The behaviour and communication of medical staff are described as having a strong influence on partners’ feelings [65]. The fathers’ role in labour is also related to the support provided by the staff [56, 70]. They need the midwife’s guidance to find their role [67]. Fathers’ needs are varied, but what seems to be the most important is *information*, especially about the birth process, particularly for those who are unmarried, have a lower education status, and for first-time fathers [57]. In Eggermont et al.’s study, formal information needs were given higher priority than involvement in the birth process, but midwives were found to overlook this or give unwanted information [57]. Hildingsson et al. also found information to be a high priority among fathers with more than one child. First-time fathers in this study, however, considered it more important for the midwife to be present and supportive [58]. Premberg et al. found that fathers whose child was born by caesarean section also rated the provision of information highly [53]. The information needed by fathers relates to what is happening and how they can help [56, 65, 66, 70]. They need support in their ability to support the woman [59], for example, by the midwife showing the father how to be supportive or for the father to imitate the midwife [56]. They want to receive clear and appropriate information, and feel more confident when midwives know when and how to act as midwives themselves [62].

In addition to information, emotional support and acceptance are important factors [53]. Fathers want to feel that their presence is important [70], to be treated with respect and empathy, and to be actively involved in the decision-making process [59, 60]. Whether fathers feel supported during childbirth depends on whether they feel included as one half of the birthing couple, or whether they feel marginalised [56]. The partner needs informative and emotional support to feel calm and find their role [50]. Men want to be treated as an important part, both as an individual and as part of the birthing couple [56]. They want to be involved but also have the option of not being involved [56]. When lack of importance and support are perceived this leads to helplessness and panic and makes their supportive role more difficult [56], or leads to a more passive role [43]. However, Longworth & Kingdon show that staff behaviour or language did not affect fathers’ feelings of being on the periphery of events during childbirth [68].

Fathers frequently report low professional support [47, 54, 56–58, 76]. Fathers would have liked the midwife to be present more often and for more information about the birth process to have been provided [47]. There is a large discrepancy between the perceived reality of receiving enough information and the subjective importance of it [58]. Support options, such as holding the birthing woman were reportedly difficult to implement because of environmental factors, e.g., lack of space or convenient options [50].

In contrast, other studies report that midwifery care, opportunities for participation and decision-making were better than expected or needed [58]; staff care is overwhelmingly positively viewed [66], and the majority found staff helpful in answering questions and reducing anxiety [69]. The midwife’s competence, her calming manner, and communication with her were perceived as helpful [66]. Fathers do not express the need for emotional support, probably because, as mentioned above, they suppress their feelings [57].

Involvement in care, trustworthy and supportive staff [60], satisfaction with midwife presence and the provision of information are related to a positive birth experience [58]. Limited participation in the decision-making process, lack of support from staff, and lack of information are related to a negative birth experience [60]. The more informed fathers feel, the more they find that the birth was as they expected [69].

While some studies find no difference in fathers’ ages [58, 69], other studies showed that younger fathers have a greater need for emotional support and acceptance [53].

#### Becoming a father

The moment of birth is described as a life-changing and overwhelming moment characterised by feelings of love and belonging [5, 65]. The best moment of the birth experience is the physical appearance of the baby [55, 72] followed by great feelings of relief [65]. Fathers want to hold the baby in their arms as soon as possible [59] and describe witnessing the birth of the child – along with supporting the woman – as the most important reason to accompany the birth [49]. Potential emotional disconnection during pregnancy and birth can now be reconnected [68]. Birth as the beginning of fatherhood is described as a transformation [5]. The cutting of the umbilical cord is referred to as a “rite of passage”, the physical separation of mother and baby [64], or the event that makes the baby an independent person [49]. The feelings are described as much stronger than expected, the greatest event in their lives [67], wonderful and different from anything else [5]. Fathers feel the bond with the child [68], but also feel the bond as a trio [5]. The birth experience can strengthen and enhance the relationship with the woman [45, 73]. However, feelings of fear for the child and strangeness are also reported [73].

## Discussion

This scoping review presents the quantitative, qualitative and mixed-method findings on fathers’ birth experiences in the clinical setting. Four important themes were found to influence fathers’ birth experiences, enhance their well-being, and promote an active and thus more satisfying role. One theme deals with the *intense feelings* reported by the fathers after the birth. During the birth process, the fathers describe strong negative feelings, especially fear and being overwhelmed. The birth of the child, on the contrary, is described as the most beautiful event of their lives. Premberg et al. describe this essential meaning of the experience as an ‘interwoven process pendulating between euphoria and agony’ [70]. These positive feelings are part of a main theme in this review, the *becoming a father*. The remaining two themes are the *role of support* and *staff support*. These two are closely related, as staff support has a significant impact on the role of the support person.

Fenwick et al. found that the desire for support from staff was expressed in advance when men were asked about their expectations for the birth, as confirmed in this review [14]. Partners want to receive information, they want to be involved, and to be part of the process. They need trustworthy and professional staff to support them and an environment that is sensitive to their needs and supportive of their role. Men want to be treated with empathy and respect. They need support from staff to find their role. One finding from this review is that fathers put the woman’s well-being first, suppress their feelings in favour of the woman, and therefore do not ask for support from staff even when they need it. It is not yet clear exactly what partners need to be able to express their true feelings and needs, and how health professionals can be more inclusive of the father. A first step toward identifying partners’ needs and concerns has been taken with this review. Following this, a more in-depth study using validated instruments is needed to determine how staff can address these needs from the partners’ perspective.

If men do not receive support, they may take a very passive role, feel helpless and as a result not experience a positive birth. Women equally want an active partner to go through the birth process with them [39]. They need physical contact and intimacy [39, 77] and want someone emotionally close to them who gives them a sense of familiarity [30]. Women can be distracted by the need to attend to their support person’s wellbeing, as they tend to be at least peripherally aware of their partner’s activities, comfort, and mood [50]. Nielsen & Overgaard show that women’s stress and anxiety levels are lower when they do not have to worry about their partner’s wellbeing [39].

Previous studies have reported that support people felt unprepared for the intensity of the unpredictable birth process and the resulting fears regarding the health and life of the partner and child [78]. In addition, feelings of discomfort and difficulties in dealing with the woman’s pain are reported [6]. These intense emotions, especially fear and feeling overwhelmed, were confirmed by the present review. Men are mainly afraid for the woman and child and are overwhelmed by the woman’s pain. We found few studies that used established and validated instruments to measure psychological outcomes, such as anxiety and stress, in fathers [11, 52]. More research is needed if we want to prevent post-traumatic stress disorder and provide partners with a positive experience of birth care. However, this scoping review has also confirmed the positive feelings described earlier [6]. The majority of fathers report a great experience and would recommend other fathers attending the birth. Studies are required to investigate whether feelings of anxiety and overwhelm are part of the process, whether they are compensated for by the joy of having a child, or whether they are stressful and long-lasting. There is also a need for studies that investigate what key factors influence the experience of overwhelm and thus what differentiates the partners in their experience.

The evidence regarding different birth modes was inconsistent, particularly with respect to caesarean section. While in some studies caesarean section was found in general to lead to a more negative birth experience, others reported that elective caesarean section led to a positive birth experience. Many of the studies included here focussed on spontaneous vaginal births and excluded other birth modes from the outset. Studies focusing on the fathers’ experience with caesarean birth found that they want to receive information and be involved in the decision-making process [76]. What is clear from the studies included in this review is that being present at an instrumental birth leads to negative feelings and a negative birth experience for the father. A study by Hildingsson et al., focusing on couples’ experiences of instrumental birth, reports fathers feeling near panic when attending an instrumental vaginal birth [79]. Nevertheless, the extent to which partners’ needs differ across birth modes remains to be determined. Therefore, more comprehensive studies are warranted to determine the specific needs of partners in different birth processes and birth modes.

Studies focusing on a non-clinical setting were excluded from this scoping review. Fathers present during a home birth report different feelings, in particular greater active mental and physical involvement [80]. In Lindgren & Erlandsson, a father at home describes himself as an interpreter and provider of safety for the woman, whereas in a hospital birth he sees himself as a protector and guardian of the woman [81]. The home environment facilitates support, and the absence of unknown people gives the father a sense of security in supporting the woman [81]. Men find it much easier to find a supportive role at home because they can relax and engage with the woman’s needs. They feel they are in the role of host rather than guest [81]. This facilitation of the paternal role during birth is difficult to achieve in the highly medicalised obstetric setting. Studies which compare the wellbeing of fathers in the homebirth environment to that in conventional birthing rooms are needed. Studies should also find out what can be changed in the clinics so that the partner can take a confident supportive role as in home births.

The present review has some potential limitations. First, the methodological quality of the studies varied, which may have influenced the results presented here. For example, the sample size of most of the quantitative studies included was small and the questionnaires used to capture birth experiences of partners were often not validated. Second, although the focus was restricted to Western countries, comparability between different healthcare and obstetric systems is limited. Results should therefore be considered with caution. Third, some studies do not specify at what time after birth the data collection took place. Thus, no conclusion can be drawn about potential recall effects in these studies. Fourth, a number of the studies reviewed were Swedish, therefore the Swedish context may be overly represented. In Sweden, fathers’ involvement is generally considered a social ‘norm’ [82]. This may be one reason why typical male gender biases have not been addressed in the studies included in this review. Dolan & Coe suggest that men’s mental construction of appropriate support during childbirth is in conflict with traditional masculine values and men feel marginalised [12]. To compensate, men construct masculine identities during childbirth which allow them to embody a masculine form (e.g., by suppressing overwhelming feelings and hiding them from the woman giving birth and staff).

## Conclusion

It is important to consider the woman, the partner and the couple as three separate and individual units. The active role of the support person should be encouraged, as they are more likely to feel useful and thus more satisfied with the birth process. To this end, the support person needs emotional and informal support from the staff. He or she needs information to reduce fears and anxieties and instruction on how to support the woman. Given the prevailing shortage of midwives and work overload of clinical staff, the wellbeing and role of the support person should be systematically studied and promoted. Results are sometimes inconsistent and studies with validated questionnaires are lacking. Systematic research should be conducted on what support people need to achieve a positive birth experience. Further, studies should be conducted which include all support people and do not focus exclusively on fathers.

## Supplementary Information

**Additional file 1.** PRISMA_ScR Checklist.

**Additional file 2.** Critical appraisal.

## Data Availability

Data sharing is not applicable to this article as no datasets were generated or analysed during the current study.

## Abbreviations

PTSD: Post-traumatic stress disorder
PRISMA_ScR: Preferred Reporting Items for Systematic reviews and Meta-Analyses extension for Scoping Reviews
Medline: Medical Literature Analysis and Retrieval System Online
PubMed: Public Medicine
CINAHL: Cumulative Index to Nursing and Allied Health Literature
MIDIRS: Midwives Information & Resource Service
MeSH: Medical Subject Headings
STAI: State-Trait-Anxiety Inventory
