# Supplementary material for: The partner’s experiences of childbirth in countries with a highly developed clinical setting: a scoping review
Source: BMC Pregnancy Childbirth. 2022 Oct 3;22:742. doi: 10.1186/s12884-022-05014-1 (PMC9528111; doi:10.1186/s12884-022-05014-1)
Supplement: Supplementary file 2 — Additional file 2. Critical appraisal. [file 12884_2022_5014_MOESM2_ESM.docx]

Additional File 2. Critical appraisal of the included studies.

Based on: Hong Q, Pluye P, Fàbregues S, Bartlett G, Boardman F, Cargo M, et al. Mixed Methods Appraisal Tool (MMAT), version 2018. Registration of Copyright (#1148552). Canadian Intellectual Property Office, Industry Canada.
